# Detection of foci of residual malaria transmission through reactive case detection in Ethiopia

**DOI:** 10.1186/s12936-018-2537-5

**Published:** 2018-10-26

**Authors:** Endalew Zemene, Cristian Koepfli, Abebaw Tiruneh, Asnakew K. Yeshiwondim, Dinberu Seyoum, Ming-Chieh Lee, Guiyun Yan, Delenasaw Yewhalaw

**Affiliations:** 1 School of Medical Laboratory Sciences, Faculty of Health Sciences, Jimma University, Jimma, Ethiopia; 2 Program in Public Health, College of Health Sciences, University of California at Irvine, Irvine, CA 92697 USA; 3 PATH/MACEPA, Addis Ababa, Ethiopia; 4 Department of Statistics, College of Natural Sciences, Jimma University, Jimma, Ethiopia; 5 Tropical and Infectious Diseases Research Centre, Jimma University, Jimma, Ethiopia

**Keywords:** Reactive case detection, Malaria, Residual malaria transmission, Low-transmission setting, Ethiopia

## Abstract

**Background:**

Sub-microscopic and asymptomatic infections could be bottlenecks to malaria elimination efforts in Ethiopia. This study determined the prevalence of malaria, and individual and household-level factors associated with *Plasmodium* infections obtained following detection of index cases in health facilities in Jimma Zone.

**Methods:**

Index malaria cases were passively detected and tracked in health facilities from June to November 2016. Moreover, family members of the index houses and neighbours located within approximately 200 m from the index houses were also screened for malaria.

**Results:**

A total of 39 index cases initiated the reactive case detection of 726 individuals in 116 households. Overall, the prevalence of malaria using microscopy and PCR was 4.0% and 8.96%, respectively. Seventeen (43.6%) of the index cases were from Doyo Yaya *kebele*, where parasite prevalence was higher. The majority of the malaria cases (90.74%) were asymptomatic. Fever (AOR = 12.68, 95% CI 3.34–48.18) and history of malaria in the preceding 1 year (AOR = 3.62, 95% CI 1.77–7.38) were significant individual-level factors associated with detection of *Plasmodium* infection. Moreover, living in index house (AOR = 2.22, 95% CI 1.16–4.27), house with eave (AOR = 2.28, 95% CI 1.14–4.55), area of residence (AOR = 6.81, 95% CI 2.49–18.63) and family size (AOR = 3.35, 95% CI 1.53–7.33) were main household-level predictors for residual malaria transmission.

**Conclusion:**

The number of index cases per *kebele* may enhance RACD efforts to detect additional malaria cases in low transmission settings. Asymptomatic and sub-microscopic infections were high in the study area, which need new or improved surveillance tools for malaria elimination efforts.

## Background

The global pattern of malaria epidemiology has changed remarkably over the last decade. Between 2010 and 2015, malaria mortality rates declined by 35% among children under 5 years of age, with 21% fall in incidence among the population at risk [1]. Implementation of key malaria prevention and control measures have played a pivotal role in decreasing morbidity and mortality due to malaria [2, 3]. Furthermore, concerted control and elimination efforts of malaria in some countries, such as the United Arab Emirates and Sri Lanka, have resulted in malaria-free status in recent years [4, 5]. Despite the achievements gained in the control of malaria, the disease still remains a significant public health problem in many sub-Saharan African countries, including Ethiopia.

The epidemiology of malaria in Ethiopia appears unique, compared to other countries in sub-Saharan Africa, in that both *Plasmodium falciparum* and *Plasmodium vivax* coexist. While almost all cases of malaria are due to the two species, there is high spatiotemporal heterogeneity in the distribution of these parasite species. According to the 2015 National Health Sector Development Plan report [6], out of the total microscopy or rapid diagnostic test (RDT) confirmed malaria cases, 63.7% and 36.3% were due to *P. falciparum* and *P. vivax*, respectively. In a recent study done in Jimma in south-western Ethiopia, however, more than three-quarters of the cases were due to *P. vivax* [7]. *Plasmodium ovale* plays a minor role in Ethiopia, and appears to be often misdiagnosed [8].

Over the last decade, during which malaria elimination was put back on the global health agenda, morbidity and mortality due to malaria has remarkably declined in Ethiopia [9, 10]. Besides the sharp decline of malaria including from some of the historically malarious areas of the country [11], no major malaria epidemics, which usually recur every 5- to 8 years, have been reported since 2005 [12]. Implementation and scale-up of the powerful vector control interventions, including indoor residual spraying (IRS) and long-lasting insecticidal nets (LLINs) appear to have played key roles [13]. More than 17 million LLINs have been distributed in 2014/2015 alone, with cumulative number of the nets distributed since 2009 being scaled up to more than 75 million [6]. Access to malaria diagnostics and treatment has also remarkably improved over the last decade, mainly via the innovative health extension programme [14] that operates at community level.

Based on the malaria control achievements gained, and with the help of international partners, Ethiopia has set goals to eliminate malaria by 2030. However, substantial portions of human *Plasmodium* infections are asymptomatic, often remaining undetected by microscopic examination [15]. Asymptomatic infections can serve as reservoirs of infection to the vector mosquitoes [16], potentially sustaining transmission.

To further sustain control of malaria and move towards elimination, adequate detection and prompt treatment of both symptomatic and asymptomatic cases in the community is critical [17]. One of the strategies of addressing malaria cases not presenting to the health care facilities is reactive case detection (RACD) with focal test and treatment methods. Reactive case detection makes use of the spatial clustering trend of malaria carriers particularly in low endemic settings [18, 19]. Hence, in RACD, following passive case detection, household members of the index case and neighbours located at certain distance from the index household are screened. This method has been utilized in several low malaria transmission settings [20, 21], despite lack of established standard approach to the spatial range of neighbouring households to be within the screening radius.

Reactive case detection also allows detection of asymptomatic malaria infections, which play a major role in sustaining malaria transmission in low-transmission settings [22]. However, active case detection of malaria is not yet fully implemented in the routine health care system in Ethiopia. Thus, this study is aimed at detecting malaria cases using RACD in two health centres in Jimma Zone, south-western Ethiopia.

## Methods

### Study setting

**Fig. 1:**
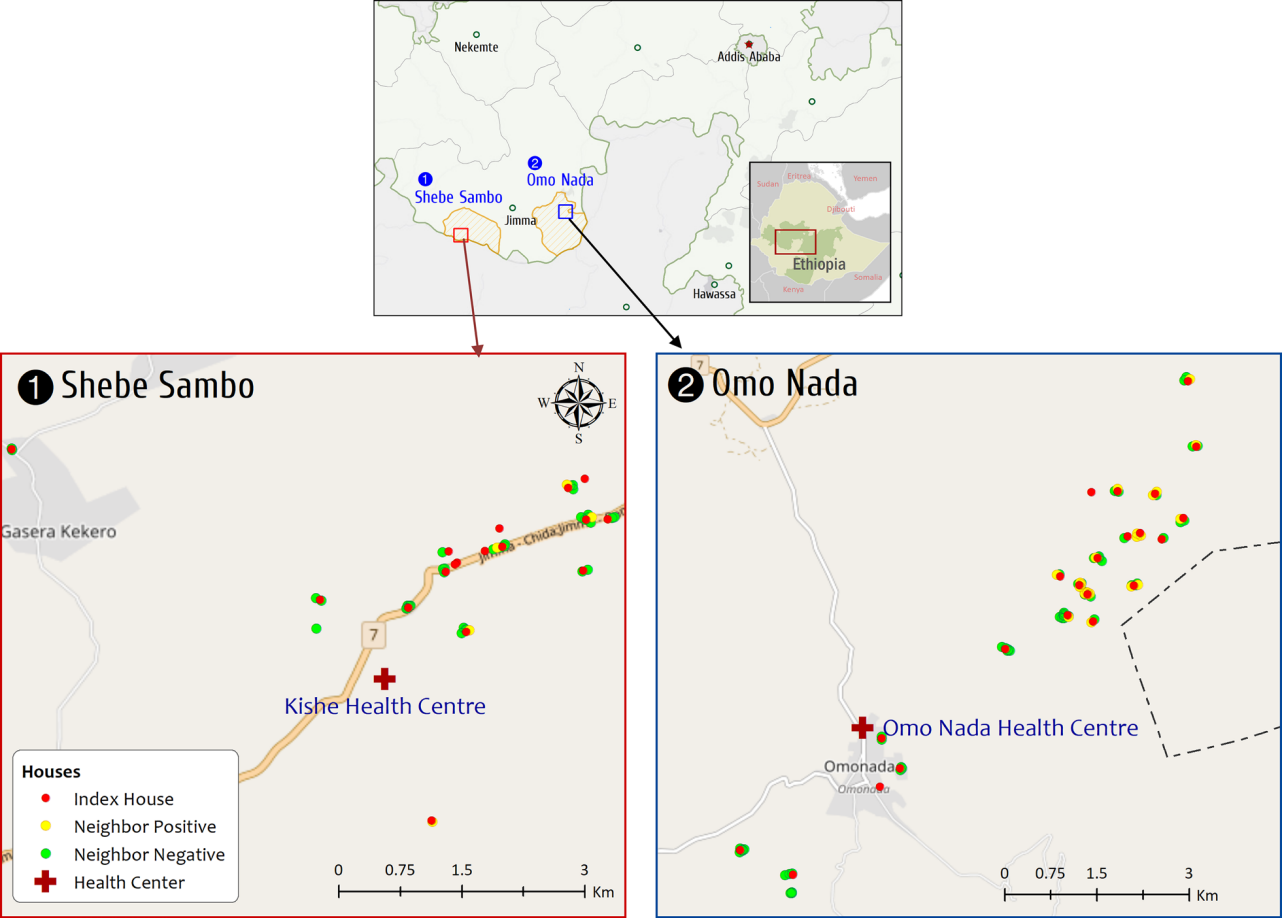
Distribution of the index houses and neighbours in the study area

The study was conducted in catchment *kebeles* (smallest government administrative units in Ethiopia) of Kishe and Nada health centres, located in Shebe Sambo and Omo Nada districts of Jimma Zone, respectively (Fig. 1). Shebe Sambo and Omo Nada districts are located at 415 and 285 kms south west of the capital, Addis Ababa, respectively. The geographical coordinates of Shebe Sambo and Omo Nada are approximately 7°30′14″N, 36°30′44″E and 7°38′00″N, 37°15′05″E, respectively. The inhabitants in both areas mainly depend on subsistence farming, cultivating mainly maize and *teff*. Moreover, in Kishe area rice is cultivated in small scale.

Historically, the catchment areas of both health centres have been malarious [23–25]. As in most parts of Ethiopia, the transmission of malaria in these areas is seasonal. The transmission usually peaks from September to October, following the major rains from June to September, and minor transmission occurs in April and May, following the short rains of February to March. According to the information obtained from both health centres, malaria cases detected in the health facilities have remarkably declined in recent years. A total 43 malaria cases have been registered in Kishe Health Centre in 2016. Of these, 62.8% were due to *P. falciparum.* Kishe Health Centre has been serving a total of 26,843 population in 2016. In the same year, a total of 51 malaria cases were recorded at Nada Health Centre, 49% of which were due to *P. falciparum*. The health centre has been serving a total of 32,264 population in 2016.

### Population and sampling

A prospective observational study was conducted for 6 months (June to November 2016) in two health centres and their catchment *kebeles*. Index malaria cases residing within the catchment *kebeles* of the two health centres who did not travel within 2 weeks prior to presenting to the health centres, and diagnosed with malaria at the health centres during the study period were included in the study. The index cases were identified in the health centres based on the routine blood film microscopy by the laboratory staff at each health facility. Following detection of the index cases, household members of the index houses and neighbours within 200 m radius were included in the study.

### Data collection

#### Passive case detection

Febrile patients who sought treatment at Kishe and Nada Health Centres from June 1 to November 31, 2016, were screened for malaria using microscopy by the resident laboratory personnel as a routine practice. Consenting index cases that were microscopy-positive and who agreed that a research team will visit them within 1 week provided their home address. To locate the index household easily, names of three nearby household heads were also recorded.

#### Reactive case detection

Presence of index cases was communicated to the research team on the same day of presentation to the health centres. The index houses and neighbours were visited within 1 week of detecting the index cases, in most cases, within 3 days. After obtaining consent, family members of the index houses and neighbours within 200 m radius from the index houses were screened for *Plasmodium* infection using RDT. Moreover, demographic information and some individual and household-level risk factors of malaria were collected using a semi-structured questionnaire. The field data was collected by experienced laboratory technologists.

The individual-level factors assessed included demographic characteristics (age, sex, educational status and occupation), recent travel history, history of malaria infection in the last 1 year, LLIN usage the previous night before the survey and axillary body temperature. Fever was defined in this study as having axillary temperature of ≥ 37.5 °C, which was measured during the survey. The household-level factors assessed included housing conditions such as roof structure, presence of visible hole on the wall, presence of eave and presence of window(s), and presence of animals within the house, family size, total number of LLINs owned during the survey and whether the house was sprayed with insecticide during the last 1 year. In Ethiopia, indoor residual spraying is performed once in a year, usually around July to September (before malaria cases peak) as transmission is mainly seasonal. Apart from the household-level characteristics, an approximate distance of each house from the index house was estimated, and coordinates of each house was taken using hand-held global positioning system unit (GPS).

All consenting members of the index houses and neighbours were enrolled in this study. Infants less than 6 months of age were not included, with the assumption that they are likely protected from malaria due to passively acquired maternal antibody, and presence of foetal haemoglobin. The household members available during the visit and those found in the nearby farming sites were included in the study. The samples were collected by a team of two laboratory technologists deployed at a time. The data collection team spent, in most cases, all the day around the index houses to maximize coverage of the screened population. However, few individuals who were not available on the day of screening around their houses were not captured, as there was no follow-up to the community members.

#### Specimen collection and laboratory processing

Finger-prick blood samples were collected from consenting study participants for blood examination by RDT and microscopy. The RDT was done for rapid diagnosis and treatment; hence, RDT-positive individuals were referred to the health centres for confirmation and treatment. Moreover, thick and thin blood smears were prepared for each study participant in the field. After air drying, the thin films were fixed with absolute methanol, and Giemsa stained at Jimma University Medical Parasitology Laboratory on the same day of collection. The slides were examined by two experienced laboratory technologists independently. Approximately 200 high-power fields of the thick blood smears were examined before declaring a microscopy-negative result. The personnel reading the slides were also blinded of the RDT results. Apart from the blood smears, three to four drops of blood were spotted on Whatman 3MM filter paper for further molecular analysis. The blood spots were air-dried and kept individually in air-tight plastic bags and stored at − 20 °C until DNA extraction.

DNA was extracted from approximately 20 µL whole blood using the QiaCube DNA extraction system as per standard protocols. DNA was eluted in 100 µL buffer, and 4 µL DNA was screened using a multiplex *P. falciparum*/*P. vivax* qPCR with limit of detection of 1 DNA copy per reaction [26]. Thus, the limit of detection of the qPCR was 1–2 parasites/µL blood.

#### Data management and analysis

Household was defined in this study as group of human subjects residing in the same house as family members. Household access to LLINs was considered “sufficient” when the ratio of the total LLINs owned by a household to the family members is at least 0.5 (assuming that one LLIN covers two individuals), and “not sufficient” when the ratio is less than 0.5 [27]. While a total of 726 individuals participated in this study providing samples for microscopic examination, sufficient DBS sample for PCR was obtained from 603 individuals. Specimens of sufficient quantity could not be obtained from the remaining individuals for PCR following blood film preparation and RDT testing, the results of which were used for immediate care. Hence, the data analysis was based on the PCR-run samples. Asymptomatic malaria infection was considered when an individual who did not experience fever at the time of the survey (axillary body temperature is less than 37.5 °C) and no malaria-related symptoms was positive for *Plasmodium* species by PCR.

The collected data were coded, entered into Excel (Microsoft Office 2010) and cleaned. The data were analysed using a statistical software package STATA 12 (StataCorp., TX, USA). Descriptive statistics including frequency, percentages and median were calculated to summarize demographic profile of the study participants. Univariate and multivariate logistic regressions were employed to determine individual and household-level factors associated with malaria infection. Multi-level regression model (mixed-effects logistic regression) was utilized to determine predictors of malaria infection among individual and household-level variables. Variables with significant association with malaria infection by the univariate analysis and those with *p* values less than 0.2 were candidates for the multivariate analyses. Odds ratio and the corresponding 95% confidence intervals were calculated to show the strength of the association. Statistical significance was set at p < 0.05 during the analysis.

## Results

### Socio-demographic characteristics

Thirty-nine passively detected microscopy-positive malaria cases (index cases) initiated the RACD. Most of the index cases (56.4%, n = 22) were from Nada Health Centre, with the remaining 17 (43.6%) being from Kishe Health Centre. Twenty-four (61.5%) and 15 (38.5%) of the index cases were due to *P. falciparum* and *P. vivax* infections, respectively. Following detection of the index cases, a total of 726 individuals residing in 116 households were screened for *Plasmodium* infection. Most of them were female (55.6%, n = 404), and the median age was 16 years (range 1 to 80 years). Demographic characteristics of the study participants are presented in Table 1.

**Table 1:** Demographic characteristics of the study participants

Characteristics	Frequency, n (%)
Age group in years	
< 5	144 (19.8)
5–15	221 (30.4)
> 15	361 (49.7)
Sex	
Male	322 (44.4)
Female	404 (55.6)
Site	
Kishe	220 (30.30)
Nada	506 (69.69)
Educational status	
Illiterate	235 (42.6)
Literate	316 (57.4)
Occupation	
Farmers	155 (21.4)
Housewives	143 (19.7)
Students	214 (29.5)
Others	38 (5.2)
House structure	
Iron sheet	496 (68.3)
Thatched	230 (31.7)
Spray status of the house	
Not sprayed	417 (57.4)
Sprayed	309 (42.6)

### Prevalence of malaria

The overall prevalence of malaria from the RACD by microscopy and PCR was 4.0% (29/726) and 8.96% (54/603), respectively. Majority of the PCR-detected cases (92.59%, n = 50) were *P. falciparum*, the remaining 4 (7.41%) being *P. vivax*. The prevalence of malaria among individuals residing in the index houses and neighbours was 17.5% and 6.83%, respectively. The majority (90.74%, n = 49) of malaria cases detected in the RACD were asymptomatic. A relatively larger number of the malaria cases were detected in Doyo Yaya *kebele* (Table 2).

**Table 2:** Residence of the index cases and frequency of individuals screened following the index cases, Jimma, south-western Ethiopia

Health facility	*Kebele*	Index cases, n (%)	Examined by PCR, n (%)	Ratio of # screened to index cases	Additional RACD cases, n (%)
Kishe Health Centre	Kishe	7 (17.9)	59 (9.8)	8.4	1 (1.7)
	Halo Sebaka	7 (17.9)	91 (15.1)	13.0	4 (4.1)
	Gasera Kekero	1 (2.6)	6 (1.0)	6.0	0 (0)
	Wala Kela	1 (2.6)	7 (1.2)	7.0	0 (0)
Omo Nada Health Centre	Doyo Yaya	17 (43.6)	375 (62.2)	22.1	49 (13.1)
	Nada	4 (10.3)	18 (3.0)	4.5	0 (0)
	Nada Chala	1 (2.6)	24 (4.0)	24.0	0 (0)
	Nada Sekote	1 (2.6)	23 (3.8)	23.0	0 (0)
Total		39 (100)	603 (100)	15.5	54 (8.96)

### Factors associated with *Plasmodium* infection

Bivariate analysis of individual and household-level factors associated with malaria positivity is shown in Table 3. The analysis revealed that several individual-level factors were significantly associated with *Plasmodium* infection, including age (COR = 1.99, 95% CI 1.06–3.74), self-reported history of malaria infection in the last 1 year before the survey (COR = 3.71, 95% CI 1.94–7.07) and fever (COR = 6.12, 95% CI 1.97–18.98). The study participants were asked if they travelled to other villages in the preceding 2 weeks before the survey. Only 3 (0.5%) reported travel and none of them were positive.

**Table 3:** Bivariate analysis results showing individual and household-level factors associated with *Plasmodium* infection among the study participants, Jimma, south-western Ethiopia

Variables	Examined by PCR, n (%)	Positive, n (%)	p-value	COR (95% CI)
Age (years)				
< 5	117 (19.40)	11 (9.40)	0.336	1.46 (0.68–3.14)
5–15	185 (30.68)	23 (12.43)	0.032	1.99 (1.06–3.74)*
> 15	301 (49.92)	20 (6.64)	Ref	
Sex				
Male	270 (44.78)	24 (8.89)	Ref	
Female	333 (55.22)	30 (9.01)	0.959	1.01 (0.58–1.78)
Malaria in the preceding 1 year				
Yes	72 (11.94)	16 (22.22)	0.001	3.71 (1.94–7.07)*
No	531 (88.06)	38 (7.16)	Ref	
Fever at the time of survey				
Yes	14 (2.32)	5 (35.71)	0.002	6.12 (1.97–18.98)*
No	589 (97.68)	49 (8.32)	Ref	
ITN usage the preceding night				
Yes	243 (40.30)	18 (7.41)	Ref	
No	360 (59.70)	36 (10.00)	0.276	1.39 (0.77–2.51)
Roof structure				
Iron sheet	398 (66.00)	32 (8.04)	Ref	
Thatched	205 (34.00)	22 (10.73)	0.274	1.38 (0.78–2.43)
Visible hole on house wall				
No	418 (69.32)	36 (8.61)	Ref	
Yes	185 (30.68)	18 (9.73)	0.658	1.14 (0.63–2.07)
Presence of window				
No	210 (34.83)	16 (7.62)	Ref	
Yes	393 (65.17)	38 (9.67)	0.402	1.30 (0.71–2.39)
Eave gap				
Absent	450 (74.63)	37 (8.22)	Ref	
Present	153 (25.37)	17 (11.11)	0.281	1.40 (0.76–2.56)
Area of residence				
Doyo Yaya	375 (62.19)	49 (13.07)	0.001	6.70 (2.63–17.08)*
Others**	228 (37.81)	5 (2.19)	Ref	
Spray status of the structure				
Not sprayed	372 (61.69)	49 (13.17)	0.001	6.86 (2.69–17.48)*
Sprayed	231 (38.31)	5 (2.16)	Ref	
Animals sleeping in the house				
No	291 (48.26)	25 (8.59)	Ref	
Yes	312 (51.74)	29 (9.29)	0.762	1.09 (0.62–1.91)
Individual residing in				
Index house	120 (19.90)	21 (17.50)	0.001	2.89 (1.60–5.21)*
Neighbour	483 (80.10)	33 (6.83)	Ref	
*Plasmodium* species of the index house				
*P. vivax*	197 (32.67)	9 (4.57)	Ref	
*P. falciparum*	406 (67.33)	45 (11.08)	0.011	2.60 (1.25–5.44)*
Family size				
Less than five	279 (46.27)	11 (3.94)	Ref	
Five or more	324 (53.73)	43 (13.27)	0.001	3.73 (1.88–7.38)*
Access to ITN				
Sufficient	217 (35.99)	11 (5.07)	Ref	
Not sufficient	386 (64.01)	43 (11.14)	0.015	2.35 (1.18–4.65)*

*COR* crude odds ratio, *ref* reference

* Significant at p < 0.05, ** includes all the remaining catchment *kebeles* of the selected health centres

Among the household-level factors assessed in this study, IRS (COR = 6.86, 95% CI 2.69–17.48), living within index house (COR = 2.89, 95% CI 1.60–5.21), area of residence (COR = 6.70, 95% CI 2.63–17.08) and family size (COR = 3.73, 95% CI 1.88–7.38) were significant risk factors of malaria.

Long-lasting insecticidal nets ownership (proportion of households who reported to have at least one LLIN) was 90.9%, but 56.82% of the households having insufficient LLINs. Bivariate analysis showed that individuals living in households with insufficient access to LLINs were at higher risk of *Plasmodium* infection (COR = 2.35, 95% CI 1.18–4.65).

Table 4 shows predictors of *Plasmodium* infection. After adjusting for other variables, fever (AOR = 10.13, 95% CI 2.76–37.16) and history of malaria (AOR = 3.97, 95% CI 1.96–8.04) were the main individual-level predictors of *Plasmodium* infection. Moreover, household-level predictors of *Plasmodium* infection include area of residence (AOR = 6.81, 95% CI 2.49–18.63), living in index house (AOR = 2.22, 95% CI 1.16–4.27), eaves (AOR = 2.28, 95% CI 1.14–4.55) and family size (AOR = 3.35, 95% CI 1.53–7.33).

**Table 4:** Results of mixed-effects logistic regression analysis of individual and household-level factors showing main predictors of *Plasmodium* infection, Jimma, south-western Ethiopia

Characteristics	Examined by PCR, n (%)	Positive, n (%)	AOR (95% CI)	p-value
Fever at the time of survey				
Yes	14 (2.32)	5 (35.71)	12.68 (3.34–48.18)	0.001*
No	589 (97.68)	49 (8.32)	Ref	
Malaria history in the preceding 1 year				
Yes	72 (11.94)	16 (22.22)	3.62 (1.77–7.38)	0.001*
No	531 (88.06)	38 (7.16)	Ref	
Area of residence				
Doyo Yaya	375 (62.19)	49 (13.07)	6.81 (2.49–18.63)	0.001*
Others**	228 (37.81)	5 (2.19)		
Individual residing in				
Index house	120 (19.90)	21 (17.50)	2.22 (1.16–4.27)	0.016*
Neighbour	483 (80.10)	33 (6.83)	Ref	
Family size				
< 5	279 (46.27)	11 (3.94)	Ref	
≥ 5	324 (53.73)	43 (13.27)	3.35 (1.53–7.33)	0.003*
Eave				
Absent	450 (74.63)	37 (8.22)	Ref	
Present	153 (25.37)	17 (11.11)	2.28 (1.14–4.55)	0.020*

*AOR* adjusted odds ratio

* Significant at p < 0.05 ** includes all the remaining catchment *kebeles* of the selected health centres

## Discussion

This study revealed that index cases presenting to health facilities enabled detection of good number of additional asymptomatic malaria in the community. Thus, screening of individuals using PCR following index malaria cases presenting to health facilities is a valuable method for detecting additional malaria cases in low transmission settings. However, it should be noted that PCR-based malaria detection techniques are largely restricted to research settings, and not currently in use by the National Malaria Control Programme in Ethiopia. This calls for evaluation of affordable alternative test methods with better sensitivity compared to microscopy to be utilised in elimination settings.

The overall prevalence of malaria detected using the RACD was 8.96%, more than 90% of the cases being due to *P. falciparum*. This is in contrast to the recent report from Jimma, in which less than a third of the malaria cases were due to *P. falciparum* [7], and the national report (63.7% of the documented malaria cases being caused by *P. falciparum*) [6]. There might be high spatio-temporal variation of *Plasmodium* species in a country, and even among villages within the same district, mainly due to difference in ecological, climatic and socio-economic factors, and interventions carried out to control malaria [28, 29]. Significant clustering of *Plasmodium* infection between *kebeles*, and households was observed in this study. The vast majority of cases were found in one of the eight *kebeles* included in this study, and individuals residing within the index house were at twofold higher risk of *Plasmodium* infections as compared to their neighbours. This has an important implication in prioritizing resources for targeted malaria control and elimination efforts in low transmission setting.

Apart from household-level clustering of the malaria cases, significantly higher prevalence of malaria was documented in Doyo Yaya *kebele* compared to other *kebeles*. Heterogeneity in transmission of malaria is a well-known feature [30]. It appears that the clustering of malaria in this *kebele* is likely due to presence of a village not covered in the preceding IRS operation. Indeed, most of the houses which did not receive IRS within the preceding 12 months of the survey were located in this *kebele*. Malaria control interventions in resource-limited areas should, therefore, target such hotspots that may contribute to sustained transmission, and possibly fuel epidemics in such settings. It is worth noting that screening of malaria was limited to approximately 200 m from index houses in this study, and thus the overall prevalence in the population was not known. Several studies utilized different screening radii around the index houses, often detecting more cases within the index household and among those residing closer to the index houses [20, 31]. However, the optimum screening radius from the index house that should be included during RACD still remains unclear. It appears that the screening radius is mainly influenced by the local epidemiology of malaria and available resources to implement RACD [32].

Improved housing structure is essential in deterring endophagic/endophilic mosquitoes from reaching the occupants [33], thus possibly reducing vector-human contact. In this study, individuals residing in houses with open eaves were significantly more likely to succumb to malaria compared to those living in houses with closed eaves. Significantly higher odds of malaria in houses with open eaves was also reported previously [34, 35]. Earlier reports around the study area indicate that *Anopheles gambiae* sensu lato (presumably *Anopheles arabiensis*), the primary malaria vector in Ethiopia, to be predominantly endophagic [36], which may explain the observed higher prevalence of malaria in those living in houses with open eaves. Significantly higher number of indoor-resting malaria vectors were also observed in houses with open eaves in a previous study from central Ethiopia [37].

The vast majority of the malaria cases detected in this study were asymptomatic, most of which were sub-microscopic. Reports also show that in low-transmission settings, asymptomatic infections are common and most of the asymptomatic infections are sub-microscopic [22, 38]. As asymptomatic malaria carriers apparently do not seek treatment, they may serve as reservoirs of infection [39], jeopardizing elimination efforts. The challenges asymptomatic malaria infections pose to the malaria elimination efforts is also exacerbated by the diagnostic limitations in detecting the infections [40]. In very low transmission settings, sub-microscopic carriers may contribute up to 50% of human-to-mosquito transmissions [41]. Apart from the possible contribution to onward transmission, persistent asymptomatic infections may be associated with other deleterious health outcomes, such as anaemia [42].

In the current study, it was also found that self-reported history of malaria in the preceding 1 year before the survey was significantly associated with *Plasmodium* infection, after adjusting for the other variables. Individuals with history of malaria were more than three times likely to have *Plasmodium* infection. Relapse with vivax malaria is well known, hence, vivax cases could be due to relapse, even if small number of *P. vivax* cases were detected. It could also be attributed to persistent *P. falciparum* asymptomatic infections. The duration of persistence of asymptomatic *P. falciparum* parasitaemia is debatable. A recent report from Cambodia shows limited duration of asymptomatic *P. falciparum* parasitaemia in low transmission setting [43], while cases of asymptomatic falciparum malaria persisting more than a decade have been documented elsewhere [44].

In this study, PCR detected 2.2-fold higher *Plasmodium* infection as compared to microscopy. This difference is comparable with other previous studies [22, 45]. As the current routine malaria diagnostic protocol in health facilities in Ethiopia involve blood film microscopy, it is inevitable that sub-microscopic infections remain a huge challenge to the envisaged malaria elimination efforts. Thus, alternative field-friendly and more sensitive diagnostic tools such as highly sensitive RDTs (HS-RDTs) or loop-mediated isothermal amplification (LAMP) need to be evaluated and used for detection of sub-microscopic/asymptomatic malaria.

The difference in the prevalence of malaria was not significantly different among different age groups and between sexes. However, family size was a one of the main predictors. In households with five or more occupants, the risk of infection was threefold, compared to those living in families with fewer than five family members. This could be due to higher number of anopheline mosquitoes related with increased number of household occupants [46].

Long-lasting insecticidal nets were the only personal protection tools utilized by the study participants. However, the difference in prevalence of malaria among households with sufficient number of LLINs was not significant. Similarly, there was no significant difference in LLIN usage the preceding night on malaria risk. These could perhaps be due to poor integrity of the LLINs, rendering only partial protection from mosquito bite, and/or possibly inaccurate information provided on the number of nets owned and their usage. The apparent lack of association of sufficient ownership or usage of the LLINs and *Plasmodium* infection could also be related with biting activity of the anopheline mosquitoes in the area. It was reported in a neighbouring district that the peak biting time of *An. gambiae* s.l. was at the early part of the night, before 21:00 h [47], during which most of the inhabitants likely do not sleep. As the LLIN coverage was high in this study, it may have ‘herd effect’, in that, those not utilizing the nets may be protected as a result of usage of the rest [48].

Sustained residual transmission of malaria in the areas necessitates assessing vector behavioural characteristics and human activities that may contribute to the on-going transmission. As LLINs and IRS interventions target vectors which feed and rest indoors, malaria elimination using these interventions alone may not be achieved [49]. Outdoor biting by the vectors as a result of long-term use of the vector control tools, and resistance to insecticides used in IRS and treating the LLINs may contribute to sustained low transmission of malaria [50, 51]. Moreover, night-time human activities may increase malaria risk [52].

The study has the following limitations: First, due to limitation of resources, the RACD was limited to catchment *kebeles* of the two health centres. Hence, passively detected malaria cases which resided out of the catchment of the two selected health centres were not included. Second, this study did not include index cases who might have visited private clinics. However, the areas being predominantly rural, it is expected that most of the residents visit the two public health facilities for treatment of fever. Finally, while this study allowed to identify high number of *Plasmodium* infections around the index cases from one site, no individuals living > 200 m from index case houses were screened for infection. Thus, it was not known how large this cluster of transmission was.

## Conclusions

The study revealed that substantial number of malaria cases, largely asymptomatic, were detected using RACD. Reactive case detection strategies utilizing malaria diagnostic tools of high sensitivity, therefore, complement the routine passive case detection in low malaria transmission areas, and may be used in malaria elimination programme. Significant household and *kebele* level clustering of the malaria cases were observed. Moreover, history of malaria, houses with eaves and family size were risk factors to *Plasmodium* infection. Further studies incorporating greater distance from index households, and including environmental factors affecting risk of malaria infection are recommended.

## Abbreviations

DBS: dried blood spot
DNA: deoxyribonucleic acid
ITNs: insecticide-treated nets
LLINs: long-lasting insecticide-treated nets
PCR: polymerase chain reaction
RACD: reactive case detection
RDT: rapid diagnostic test
